# Molecular Pathology of Neuro-AIDS (CNS-HIV)

**DOI:** 10.3390/ijms10031045

**Published:** 2009-03-11

**Authors:** Leslie Crews, Christina Patrick, Cristian L. Achim, Ian P. Everall, Eliezer Masliah

**Affiliations:** 1 Department of Pathology, University of California, San Diego / 9500 Gilman Dr. La Jolla, CA 92093, U.S.A.; E-Mail: lcrews@ucsd.edu; 2 Department of Neurosciences, University of California, San Diego / 9500 Gilman Dr. La Jolla, CA 92093, U.S.A.; E-Mail: cpatrick@ucsd.edu; 3 Department of Psychiatry, University of California, San Diego / 9500 Gilman Dr. La Jolla, CA 92093, U.S.A.; E-Mails: cachim@ucsd.edu (C.A.); ieverall@ucsd.edu (I.E.)

**Keywords:** HIV, encephalitis, NeuroAIDS, inflammation, GSK3β, CDK5

## Abstract

The cognitive deficits in patients with HIV profoundly affect the quality of life of people living with this disease and have often been linked to the neuro-inflammatory condition known as HIV encephalitis (HIVE). With the advent of more effective anti-retroviral therapies, HIVE has shifted from a sub-acute to a chronic condition. The neurodegenerative process in patients with HIVE is characterized by synaptic and dendritic damage to pyramidal neurons, loss of calbindin-immunoreactive interneurons and myelin loss. The mechanisms leading to neurodegeneration in HIVE might involve a variety of pathways, and several lines of investigation have found that interference with signaling factors mediating neuroprotection might play an important role. These signaling pathways include, among others, the GSK3β, CDK5, ERK, Pyk2, p38 and JNK cascades. Of these, GSK3β has been a primary focus of many previous studies showing that in infected patients, HIV proteins and neurotoxins secreted by immune-activated cells in the brain abnormally activate this pathway, which is otherwise regulated by growth factors such as FGF. Interestingly, modulation of the GSK3β signaling pathway by FGF1 or GSK3β inhibitors (lithium, valproic acid) is protective against HIV neurotoxicity, and several pilot clinical trials have demonstrated cognitive improvements in HIV patients treated with GSK3β inhibitors. In addition to the GSK3β pathway, the CDK5 pathway has recently been implicated as a mediator of neurotoxicity in HIV, and HIV proteins might activate this pathway and subsequently disrupt the diverse processes that CDK5 regulates, including synapse formation and plasticity and neurogenesis. Taken together, the GSK3β and CDK5 signaling pathways are important regulators of neurotoxicity in HIV, and modulation of these factors might have therapeutic potential in the treatment of patients suffering from HIVE. In this context, the subsequent sections will focus on reviewing the involvement of the GSK3β and CDK5 pathways in neurodegeneration in HIV.

## 1. Introduction

The control and eradication of the neurological complications associated with AIDS continues to be an important goal in efforts toward improving the well-being of patients with HIV. In the central nervous system (CNS) of these patients, brain microglial cells are a primary reservoir for HIV-1 infection [1,2]. These immune-activated cells secrete neurotoxins and produce HIV-1 proteins that contribute to the development of HIV-associated cognitive impairment (HACI) [3]. This progressive neurologic disease is characterized clinically by cognitive deficits that profoundly affect the quality of life of people living with this condition. Cognitive impairment in patients with HIV infection has often been linked to HIV encephalitis (HIVE) [4], a neuro-inflammatory condition [2,5] characterized by the presence of HIV-infected microglial cells, formation of microglial nodules, multinucleated giant cells, astrogliosis, myelin loss and neurodegeneration [6,7].

With the advent of highly active antiretroviral therapies (HAART), the abundance of HIV in the brain and overt dementia has declined, however as the number of treated subjects with chronic HIV infection increases, the prevalence of HACI is actually rising, despite HAART [8–11]. It is now becoming apparent that these patients may be suffering from protracted forms of HIVE [7,8] that might lead to more subtle cognitive alterations rather than to overt dementia [1,4,12,13]. Patients with protracted mild forms of HIVE sometimes display more severe neurodegenerative pathology characterized by the simplification of the synapto-dendritic structure of pyramidal neuronal populations in the neocortex, indicating that HIVE has transitioned from a subacute to a chronic condition [6]. Thus, identification of new targets that might protect the CNS from the toxic effects of HIV might be an important adjuvant therapy for patients with HACI.

## 2. Molecular and cellular mechanisms of neurodegeneration in HIV Encephalitis

The mechanisms leading to cognitive impairment and dementia in AIDS patients are complex and require further investigation. Various line of evidence indicate that when HIV-infected monocytes/macrophages activate neuro-inflammatory cells such as microglia and astrocytes [14–21], these cells produce chemokines, cytokines and neurotoxins that, in conjunction with secreted HIV proteins, damage the synapto-dendritic arbor of neurons [22], leading to neuronal dysfunction and cell death probably via apoptosis [23–32]. This model predicts that levels of HIV in the CNS might reflect the extent of the structural and functional pathology in the brain [33–35]. Most patients with florid HIVE display cognitive impairment that in some severe cases results in dementia [4]. However, about a quarter of patients with evidence of HIVE at autopsy clinically are neurologically un-impaired. Most cases with no significant neuropathology are cognitively un-impaired, nonetheless approximately one third of cases with no evidence of HIVE display cognitive alterations [4].

**Figure 1.**
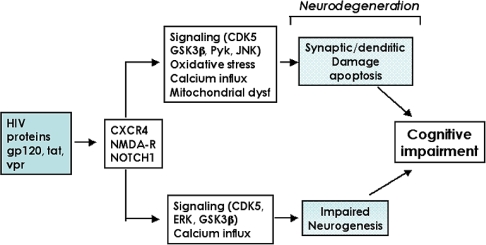
Mechanisms of HIV protein-induced neuronal damage. HIV protein-mediated activation of cell surface receptors (CXCR4, NMDA-R, Notch 1) leads to alterations in signaling pathways such as GSK3β and CDK5. Under physiological conditions, these pathways modulate critical substrates involved in synaptic plasticity and neurogenesis, and dysregulation of these signaling cascades could result in synapto-dendritic damage and impaired neurogenesis.

The neurodegenerative process in patients with HIVE is characterized by synaptic and dendritic damage [36] to pyramidal neurons [36], loss of calbindin-immunoreactive interneurons [37] and myelin loss [38]. While disruption of the cortico-cortico connections might result in learning and attention deficits, hippocampal pathology is linked to memory loss, and cortico-striatal damage results in motor alterations [39]. In addition to the damage to mature neuronal circuitries, recent studies have shown that HIV proteins might contribute to the neurodegenerative process by interfering with neurogenesis in the hippocampus [40–43]. Neurogenesis in the dentate gyrus is an active process in the mature CNS and plays a key role in synaptic plasticity, memory, and learning [44,45]. Environmental enrichment has been shown to stimulate neurogenesis and improve performance in memory tasks in mice [46–48]. The wnt [49] GSK3β and the cyclin-dependent kinase (CDK) signaling pathways [50] play an important role in this process. Therefore, the neurodegenerative process leading to cognitive alterations in HIV patients includes both: **1)** damage to the mature synapto-dendritic apparatus of developed neurons and **2)** impaired ability of NPCs in the hippocampal dentate gyrus to generate new neurons (Figure 1).

The mechanisms leading to neurodegeneration in HIVE might involve a variety of pathways including excitotoxicity [51,52], oxidative stress [23], mitochondrial dysfunction [53,54] and calcium dysregulation [55,56] (Figure 1). In addition, several lines of investigation have found that interference with signaling pathways mediating neuroprotection might also play an important role. Among them, previous studies have shown that HIV proteins abnormally activate the CDK5 [57,58], GSK3β [59,60] and ERK [61,62] signaling pathways, which otherwise are regulated by fibroblast growth factors (FGFs) [63,64]. In addition to direct activation of these signaling pathways, HIV proteins have been shown to trigger the release of neurotoxic products such as platelet-activating factor (PAF), which also contribute to dysregulation of signaling pathways such as GSK3β [65]. Furthermore, HIV proteins trigger neurodegeneration by activating signaling pathways involved in apoptosis such as RNA-activated protein kinase [66], Pyk2 [67], p38 and INK [24,61,68]. Of these molecular pathways leading to neurodegeneration in patients with HIVE, the subsequent sections will focus on reviewing the involvement of the GSK3β and CDK5 pathways.

### 2.1. Role of FGF1-mediated regulation of the GSK3β signaling pathway in the mechanisms of neurodegeneration and neuroprotection in HIVE

Previous studies found that in patients with cognitive impairment and HIVE, neurodegeneration might be associated with a reduction in the expression of neurotrophic factors such as FGF1 [69]. In contrast, in a subset of patients with HIV with preserved cognitive performance, FGF1 levels are up-regulated. Interestingly, neuronal populations prone to degenerate in patients with HIV are dependent on FGF1 and FGF2 and express FGF receptors and CXCR4 [6,63, 70]. The FGFs belong to a family 13 trophic factors that are involved in neurogenesis [71] and angiogenesis [72]. Since FGF1 and 2 are expressed in the CNS in neurons vulnerable to the neurotoxic effects of HIV proteins [69], this indicates that the molecular pathways modulating FGF1 effects might be important mediators of the signaling involved in neurodegeneration and neuroprotection in patients with HIV [73].

*In vitro* studies in primary neurons and neuronal cell lines have shown that the neuroprotective effects of FGF1 and FGF2 are mediated by activation of PI3K-Akt that in turn inactivate GSK3β via phosphorylation at the Ser 9 residue [63,64]. In addition to FGF1 and FGF2, other growth factors that exert their effects via receptor tyrosine kinases also lead to inactivation of GSK3β through phosphorylation. These include growth factors such as insulin growth factor-1 (IGF-I), epidermal growth factor (EGF) and platelet-derived growth factor (PDGF) [74,75]. To further investigate the neuroprotective effects of GSK3β regulation by FGF1 *in vivo,* we generated lines of tg mice expressing the human FGF1 under a neuronal promoter (PDGFβ).

**Figure 2.**
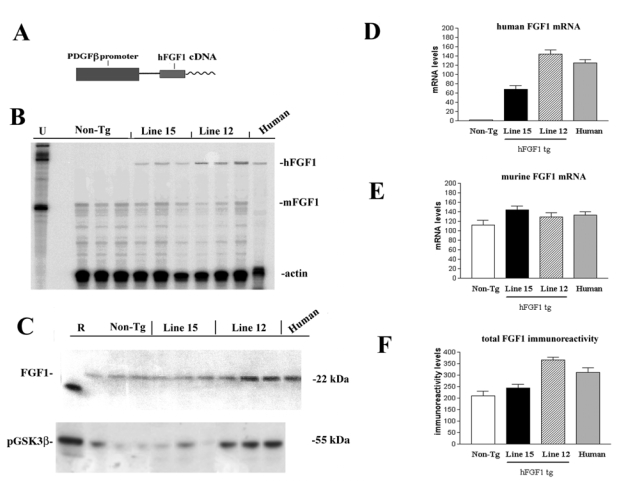
Characterization of hFGFl tg mice, (a) Construct expressing hFGFl under the control of the PDGFβ promoter, (b) RPA analysis of FGF1 mRNA expression, (c) Immunoblot analysis of total FGF1 protein expression and inactivation of GSK3β in FGF1 tg mice, (d) Semi-quantitative analysis of hFGFl mRNA levels, (e) Semi-quantitative analysis of mFGFl mRNA levels, (e) Semi-quantitative analysis of total FGF1 protein expression by immunoblot.

Human FGF1 cDNA was obtained by reverse transcriptase polymerase chain reaction (RT-PCR) from human brains and cloned into PCRII vector (TA Cloning from Invitrogen, CA) and 100% fidelity of nucleotide sequence was confirmed by dideoxy sequencing. Subsequently the FGF1 cDNA fragment was subcloned into the PDGFβ transgene cassette. The PDGFβ promoter was a gift of Dr. Tucker Collins at Harvard Medical School. The final construct contains the PDGFβ promoter, SV40 intron, hFGFl cDNAs, and SV40 polyA (Figure 2A). Constructs were microinjected and 5 lines of founder mice were obtained. Of them, based on the levels of mRNA expression two transgenic lines (line 15 low expresser; line 12 moderate expresser) were selected. RPA and Western blot analysis showed that both lines expressed human (h)FGFl at levels comparable to the levels in the human brain (Figure 2B–F). Immunocytochemical analysis confirmed that hFGFl was primarily expressed by neurons in the neocortex, hippocampus and basal ganglia, regions selectively susceptible to the neurotoxic effects of HIV products. Both lines of hFGFl tg mice were viable, bred well and the nervous system developed normally. To determine the effects of FGF1 expression on the GSK3β signaling pathway, immunoblot analysis was performed with an antibody against phosphorylated GSK3β. This showed that in the mouse line expressing moderate levels of hFGFl (line 12), levels of phosphorylated GSK3β (inactive form) were increased, while levels of pGSK3β in the low expresser line (15) were similar to nontg controls (Figure 2C).

**Figure 3.**
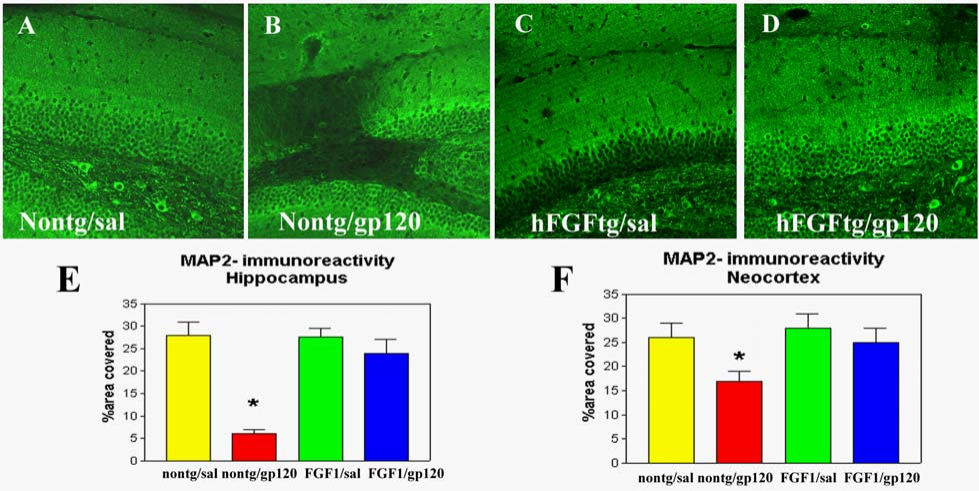
Protective effects of FGF1 expression against the toxic effects of HIV-gp120 in mice injected with gp120 protein, (a) Nontg mouse injected with saline, (b) A prominent lesion developed in nontg mice injected with gp120 protein, (c) hFGFl tg mouse injected with saline, (d) In hFGFl tg mice injected with gp120, the neurons are protected and no lesion is observed, (e) Semi-quantitative analysis of levels of MAP2 immunoreactivity in the hippocampus, (f) Semi-quantitative analysis of levels of MAP2 immunoreactivity in the cortex.

In order to test the hypothesis that hFGFl protects against the neurotoxic effects of HIV products, tg mice (3 mo old, 5 per group) from lines 12 and 15 received intracerebral gp120 injections (lmM, total 2μl) in the neocortex and hippocampus. In nontg mice (3 mo old, 5 per group), gp120 promoted significant neuronal damage and astrogliosis compared to nontg saline-treated mice (Figure 3). In hFGFl tg mice from line 12 (moderate expresser) neurons were protected against the toxic effects of gp120, while line 15 mice (low expresser) were susceptible, supporting the contention that hFGFl was bioactive and protected neurons in a dose-dependent manner, probably via inactivation of GSK3β. Therefore, treatments directed at increasing the expression of FGF1 or targeting the signaling pathways affected, such as GSK3β, might represent a possible neuroprotective strategy.

### 2.2. Neuroprotective effects of GSK3β inhibition in HIV neurotoxicity

Similar to FGF1-mediated neuroprotection against HIV toxicity, recent studies have shown that at low concentrations lithium—a GSK3β inhibitor—protects the CNS from toxins [76,77] via GSK3β inactivation. Our previous studies have shown lithium pre-treatment is neuroprotective in mice that received intrahippocampal injections of recombinant gp120 protein [76]. To expand on these studies, and to investigate the protective effects of lithium-mediated regulation of GSK3β against HIV-gp120-mediated toxicity in a tg model, mice expressing high levels of gp120 in the brain were treated with lithium and analyzed by immunocytochemistry and immunoblot. For this purpose, 4-month old gp120 tg mice (n=6) received intraperitoneal injections of lithium chloride (2 mg/kg/day) (Sigma, St. Louis, MO) for 14 days, and 6 control mice received saline injections (100μl/day for 14 days).

**Figure 4.**
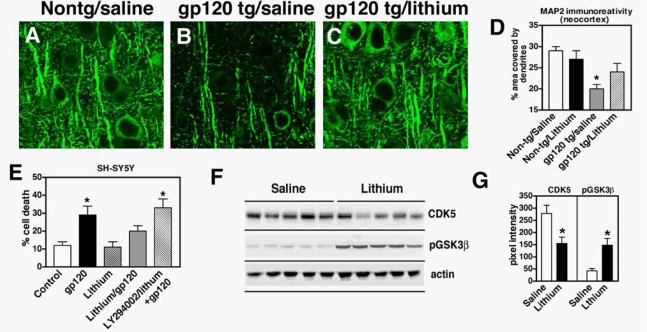
Neuroprotective effects of lithium in gp120 models via GSK3β and CDK5 phosphorylation. (a-d) Amelioration of the dendritic pathology (MAP2) in gp120 tg mice treated with lithium (daily injections for 2 weeks), (e) The neuroprotective effects of lithium against gp120 in SH-SY5Y cells are mediated by Akt. (f, g) Lithium downregulates CDK5 hyperactivation and inactivates GSK3β via phosphorylation.

Consistent with our previous study in lithium-treated mice that received intrahippocampal injections of gp120 [76], lithium treatment in gp120 tg mice protected the hippocampus of mice from gp120-mediated toxicity (Figure 4) [76]. Lithium ameliorated the dendritic damage in gp120 tg mice (Figure 4A-D). Similarly, pre-exposure of neuronal cultures to lithium significantly reduced gp120-associated neurotoxicity [76] (Figure 4E). The protective effects of lithium *in vitro* were partially blocked by LY294002, an inhibitor of the PI3K/Akt/GSK3β pathway (Figure 4E). Immunoblot analysis showed that lithium activates Akt and blocks GSK3β activity via phosphorylation at Ser 9 (Figure 4F). This is consistent with previous studies showing that lithium and other inhibitors of GSK3β activation, such as valproic acid, protect the brain against HIV-associated neurotoxicity [78–82]. Therefore, the neuroprotective effects of FGF1 and lithium or valproic acid involve targeting analogous pathways, namely the Akt/GSK3β signaling cascade.

In view of this evidence and previous studies showing that GSK3β activation is involved in the mechanisms of HIV neurotoxicity [60], we concluded that targeting the GSK3β pathway with lithium might be of value in the management of the cognitive impairment [76,81–83]. Remarkably, in a pilot clinical trial we found that treatment with lithium for 12 weeks significantly ameliorated the cognitive deficits in HIV patients [83]. Similarly, another pilot clinical trial demonstrated that valproic acid is well tolerated in patients and can ameliorate HACI [78,80]. Taken together, these studies support a role for the GSK3β signaling pathway as a potential therapeutic target in the treatment of neuroAIDS.

Currently, we are in the process of extending the lithium studies as well as investigating the potential therapeutic value of analogous molecules and signaling targets. In this regard, it is becoming clear that the neuroprotective effects of FGF1 and lithium might involve regulation of other signaling cascades [84]. Immunoblot analysis of brain homogenates from gp120 tg mice treated with saline or lithium showed that in addition to activating Akt and blocking GSK3β, lithium treatment also resulted in reduced levels of CDK5 expression (Figure 4F), but no effects were observed on p38 and SAPK. These findings support the contention that both the PI3K/Akt/GSK3β and the CDK5 signaling pathways might be involved in mediating neurotoxicity in HIVE, and that regulation of CDK5 in pathological states might be an important target for neuroprotection.

### 2.3. Role of the CDK5 pathway in the mechanisms of synaptic damage in HIVE

**Figure 5.**
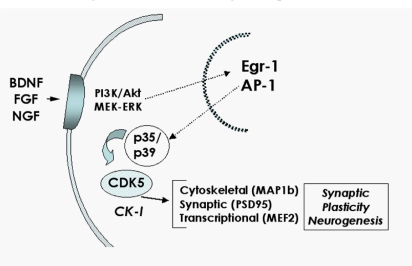
Physiological role of p35-CDK5 signaling in neurons. In the brain, CDK5 is the predominant CDK and can be activated by p35/p39 and phosphorylation of p35 by CK1. CDK5 activation, in turn, results in phosphorylation of key substrates involved in synaptic plasticity (PSD95) and neurogenesis (DCX), among other processes.

Recently, and as part of a gene array study, we found that several components of the CDK5 signaling pathway are altered in patients with HIVE [85]. Moreover, other studies have shown that activation of CDK5 by calpains contributes to HIV-induced neurotoxicity [58]. In the mature CNS CDK5 is the predominant CDK, and is expressed at high levels in neurons [86]. While in peripheral dividing tissues other CDKs function as regulators of the cell cycle, CDK5 is unique among the CDKs in that its primary role is in regulating the phosphorylation of a multitude of downstream targets involved in cytoskeletal and synaptic function, among other activities [87]. Through its diverse targets, CDK5 plays an important role in critical processes in both the mature and developing brain, including neurogenesis [50,88], neuronal migration [89], and synapse formation and plasticity [87,90,91] (Figure 5).

CDK5 is a Ser-Thr protein kinase with postmitotic activity that phosphorylates KSP motifs on cytoskeletal proteins (MAPlb, tau, NF, nestin, DCX), synaptic proteins (PSD95, synapsin, cadherin) and transcription factors (MEF2) [92–94]. While in dividing neurons CDKs are activated by cyclins, in the nervous system CDK5 is activated by forming a complex with p35 or p39 [93,95] (Figure 5). In contrast to other CDKs, which are inhibited by the cell cycle regulator proteins p21 and p27 (Kip-1) [96], CDK5 activity is regulated primarily by the metabolism of the activating proteins p35 and p39 [97].

Regulated activation of the CDK5 pathway via p35 [98] plays an important role in neuronal development [99] and synaptic plasticity [86,91] (Figure 5). The p35/p39-CDK5 complex has been shown to be constitutively active, however CDK5 phosphorylation by casein kinase 1 (CK1) [100,101] and CDK7 [102] at Ser159 also contribute to CDK5 activation. CDK5 can be further activated by phosphorylation at Tyr15 by Src-related tyrosine kinase [100]. However, it is unclear to what extent phosphorylation plays a role in the regulation of CDK5 activity, and most studies suggest that the primary mechanism controlling CDK5 activation is expression and metabolism of p35 [97].

**Figure 6.**
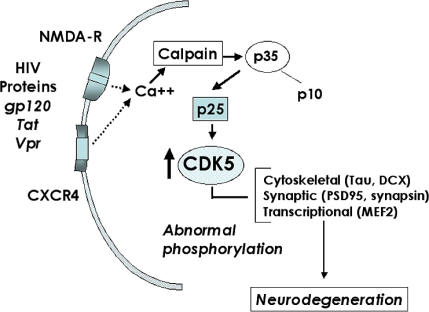
Potential mechanisms of HIV-induced neurodegeneration mediated by hyperactivation of CDK5. Activation of cell surface receptors by HIV proteins or cellular neurotoxins causes calcium influx, which might activate calpain and result in p35 cleavage into p25, which in turn hyperactivates CDK5. This leads to aberrant phosphorylation of CDK5 substrates involved in synaptic plasticity (PSD95, synapsin) and neurogenesis (DCX). Alterations in these critical functions might contribute to the underlying neurodegenerative process of HACI.

Synthesis of p35 is stimulated by NGF and BDNF [103] as well as by ECM molecules like laminin [104] (Figure 5). Interestingly, HIV-1 Tat inhibits NGF-induced Egr-1 transcriptional activity and consequent p35 expression in neural cells [57]. The phosphorylation of p35, which is catalyzed by CDK5, produces an upward shift observed by gel electrophoresis and can be utilized as an indicator of CDK5 activity [100]. Although regulated activation of CDK5 by p35 and p39 under physiological conditions promotes neuronal development [99], axonal transport and synaptic activity [86,87,91], recent evidence suggests that abnormal activation of the CDK5 pathway might be also involved in cell death and neurodegeneration [105–108] (Figure 6). In this regard, recent studies indicate that calpain I mediated cleavage of p35 into the N-truncated fragmented p25 results in hyperactivation of CDK5 which in turn results in abnormal phosphorylation of toxic substrates that have been associated with cell death [106] (Figure 6).

For example, in AD, it has been proposed that amyloid-β (Aβ) protein triggers calpain-mediated cleavage of p35 into p10 and p25, which in turn results in uncontrolled activity of CDK5 and tau hyperphosphorylation [106]. Similarly, in the nervous systems of patients with AIDS, it is possible that HIV products such as gp120 might trigger calcium influx via chemokine or glutamate receptors. This, in turn, may stimulate calpain-mediated proteolysis of p35 into p25 and subsequent hyperactivation of CDK5 with abnormal substrate phosphorylation (Figure 6). Alternatively, HIV proteins might regulate CDK5 activity by sequestering p35, or interfering with factors that modulate levels of p35 expression or stability. Since CDK5 plays a role both in synaptic function and neuronal development, then abnormal activation of this molecule by HIV proteins might not only impair the functioning of mature neurons but also contribute to alterations in neurogenesis.

### 2.4. CDK inhibitors as potential neuroprotective therapies for HACI

Although HAART is an indispensable tool for the management of the neurological complications associated with HIV neuro-invasion, this approach does not completely eradicate HIV from the CNS, nor does it provide protection from the neurotoxicity of HIV proteins. Therefore discovery and testing of neuroprotective agents continues to be an important goal as an adjuvant therapy for the cognitive and neurological complications of HIV. Efforts in this direction include the testing of NMDA-R blockers such as memantine [109–111]. In addition, we have recently shown in a pilot clinical trial that modulation of signaling pathways involved in neurodegeneration with lithium might have a potential role as a neuroprotective treatment in HIV patients [83]. Lithium’s effects involve regulation of GSK3β and probably CDK5 among other potential targets [84]. Interestingly, and as indicated before, both of these signaling pathways are altered in patients with HIVE. Although lithium [76,83] and valproic acid [78,80,81] might hold some promise as therapeutic regulators of the GSK3β and CDK5 signaling pathways, it is necessary to test and develop new and more specific compounds that target CDK5.

In recent years considerable efforts have been devoted at developing CDK inhibitors for the treatment of neoplastic, viral and neurodegenerative disorders [112]. Among them, roscovitine has been identified as a CDK5 inhibitor with therapeutic potential. This compound belongs to the family of purine-derived CDK inhibitors that includes olomoucine and purvalanols [113]. Roscovitine crosses the blood brain barrier [114] and has been shown to be protective in experimental models of neurotoxicity [115]. This compound has been shown to be safe in humans and in clinical trials for glial cell tumors has been shown to have a promising effect. Roscovitine also blocks to a lesser extent cdc42, CDK2 and ERK [113]. Remarkably, CDK inhibitors are also under development as anti-retroviral agents, because HIV recruits these kinases for viral replication in mitotic cells [112]. Of the CDK inhibitors, flavopiridol, which targets CDK1, 2, 4, 7 and 9, in combination with roscovitine, appears to be effective [112]. Thus CDK inhibitors could target both the viral infection as well as the pathogenic mechanism.

## 3. Conclusions

**Figure 7.**
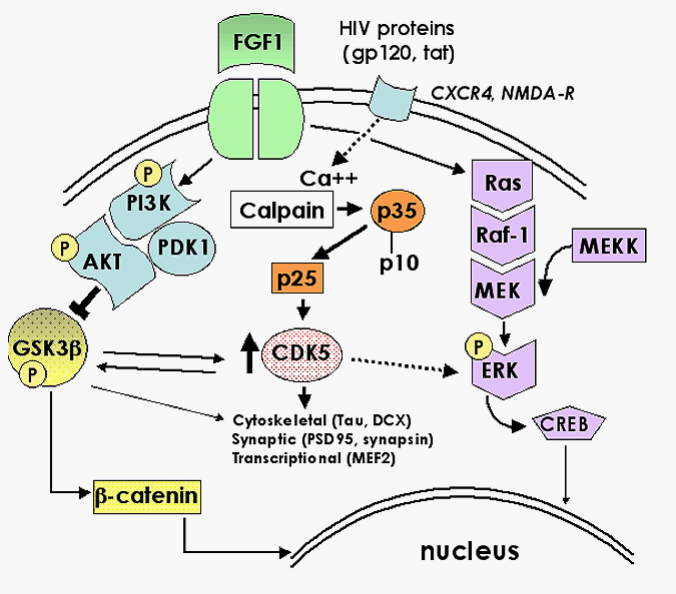
Schematic diagram showing the role of the GSK3β and CDK5 signaling pathways in mediating the toxic effects of HIV proteins. HIV proteins and cellular neurotoxins might stimulate the GSK3β and CDK5 signaling pathways via receptor interactions, resulting in the downstream disruption of processes involved in regulating synaptic and neuronal integrity. Growth factors such as FGF1, or other inhibitors of GSK3β signaling, might ameliorate these effects by antagonizing the activation of GSK3β by HIV proteins and cellular neurotoxins. GSK3β and CDK5 share several downstream targets (e.g. Tau), and simultaneous activation of both of these pathways may exacerbate the neurotoxic effects of HIV proteins.

Given that HIV persists in the CNS, then elucidating the signaling pathways involved in mediating the neurotoxic effects of HIV are critical in the development of neuroprotective therapies that might act in conjunction with anti-retroviral therapies. In this context, both the GSK3β and CDK5 signaling cascades mediate some of the neurotoxic effects of HIV proteins (Figure 7). Modulation of these pathways by growth factor (FGFl)-based approaches, or with GSK3β inhibitors (lithium, valproic acid) or CDK5 inhibitors (Roscovitine) holds promise for the development of treatments that may ameliorate the neuropathological effects exerted by HIV proteins.
